# Internet Use and Depressive Symptoms Among Chinese Older Adults: The Mediation and Suppression Effects of Social Capital

**DOI:** 10.3389/fpsyg.2021.729790

**Published:** 2021-09-21

**Authors:** Zhiyi Li, Mengyao Yang

**Affiliations:** Institute for Empirical Social Science Research, School of Humanities and Social Science, Xi'an Jiaotong University, Xi'an, China

**Keywords:** Internet use, social capital, depressive symptoms, mediating effect, suppressing effect

## Abstract

Exploring the social factors of mental health among older adults has become a hot topic. This study aimed to examine the relationships between internet use, social capital and depressive symptoms in older adults. Our data were derived from a sample of 6,840 respondents aged 60 and over in the 2018 wave of the China Family Panel Studies. The ordinary least square (OLS) regression results showed that both Internet use characteristics (including access, emotional activities, and online time) and social capital components (including contact with adult children and trust) were protective factors for the prevention of depressive symptoms among older adults. The generalized structural equation modeling (GSEM) results displayed that Internet use not only had a negatively direct effect on depressive symptoms, but also generated a negatively indirect effect on depressive symptoms by structural social capital (i.e., contact with adult children), suggesting that structural social capital mediated the above link. Conversely, the indirect effects of internet use on depressive symptoms via cognitive social capital (i.e., interpersonal trust and institutional trust) were significantly positive, indicating that the relationship between Internet use and depressive symptoms was suppressed by cognitive social capital. These findings address the gaps in previous research on older adults' mental health and have practical implications for policy makers.

## Introduction

Under the context of global population aging, mental health problems among older adults have aroused broad concern. Aging is closely associated with sensory loss, cognitive declines, and functional impairments, leading to the prevalence of late-life depression (Levy-Cushman et al., 1999). Almost 11% of older adults have depression or clinically relevant depressive symptoms (Lim et al., 2018). Geriatric depression and other health problems triggered by it, including self-harm, dementia, and suicide, not only seriously damage older adults' quality of life, but also place a heavy burden on families and countries (Luijendijk et al., 2008). Especially for older adults who live in the middle and low-income countries, their mental health could be worse due to lack of mental health resources and awareness toward the disease (Wang R. et al., 2019). For instance, studies in China found that over 90% of the older adults with noticeable depressive symptoms had never received professional treatment, and they were also reluctant to seek medical help because of a sense of “losing face” when having mental health problems (Liu et al., 2017). Therefore, it is crucial for researchers to explore a new approach that is low in cost and more socially acceptable than the traditional medication treatment to prevent depressive symptoms.

### Internet Use and Older Adults' Depressive Symptoms

As the proportion of older adults in netizens continues to rise, research into the significance of this digital technology for older adults is in the ascendant. Based on the activity theory, Internet use can mitigate the adverse effects of the lack of social activities and interactions on mental health (Lemon et al., 1972). More specifically, older adults would achieve remote communication, take part in various virtual communities, maintain their social roles in the Internet, thus enhance their sense of social connectedness and improve cognitive function, ultimately, alleviate their depression (Blit-Cohen and Litwin, 2004).

The positive role of the Internet in the mental health of older adults has been empirically tested (Cotten et al., 2014; Hofer et al., 2019). However, the precise mechanism of this relationship is not yet fully illustrated. In other words, the majority of the research mentioned above focused on the direct correlation between the two, while ignoring the mechanisms through which Internet use produced an effect on depressive symptoms.

### Social Capital and Older Adults' Depressive Symptoms

Social capital has been identified as another protective factor for mental health. There are different definitions of social capital, but it mainly refers to social networks and resources within groups or local communities, including social contact, social participation, trust, and reciprocity (Coleman, 1994; Putnam, 2012). As a multifaceted concept, social capital involves diverse directions, levels, and aspects. From the direction of social capital, it covers bonding (i.e., inner groups), bridging (i.e., between groups), and linking social capital (i.e., across social status) (Woolcock, 2001). From the level of analysis, social capital includes individual resource (i.e., personal social networks) and community resource (i.e., social cohesion, norms, as well as trust within local communities) (Bourdieu, 1986). From the attribute of social capital, it consists of structural components (i.e., social network and social participation) and cognitive components (i.e., trust) (Harpham et al., 2002). The classification standard of social capital is chiefly based on the research topic. In health-related studies, social capital is usually measured by its cognitive and structural dimensions.

Older adults surrounded by various stressful events might gain additional helps through social capital, thus buffering the negative effects of stressors like retirement or physical diseases. Specifically, trust helps older adults to shape an optimistic attitude to future, diminishing their considerable anxiety about the uncertainties of life (Economou et al., 2014). Attending organizations and social activities obviously enhance self-identification and a sense of belonging, which slow down the pace of disengagement from society for older adults (Forsman et al., 2013; Inoue et al., 2019). Informal social networks like interacting with friends or adult children could provide necessary emotional support and instrumental support when older adults need (Broese Van Groenou et al., 2013; Wang et al., 2020).

### Social Capital: The Mediating and Suppressing Role in the Link Between Internet Use and Mental Health

Very recently, a few of studies have discussed how Internet use affects depression by social capital. For instance, exploiting longitudinal mediation approach, Szabo found that using Internet could yield a positive effect on wellbeing among older adults in New Zealand by facilitating social activities (Szabo et al., 2019). One similar pathway has been verified in research targeting American older adults, which revealed that Internet use encourages frequency of interaction with acquaintances, ultimately promotes mental health (Yu et al., 2020). Conversely, other research pointed out that extending social capital through Internet might generate a distinct decline in subjective wellbeing of older adults, partly because the weak ties established from online social activities can not replace the important role of strong ties in maintaining mental health and are more likely to isolate older adults from reality (Sum et al., 2008a).

In addition, another challenge is highlighted because the impact of cognitive social capital has not yet been discussed in prior studies that only focused on the pathway of structural components. Actually, cognitive social capital has been considered as a more essential factor than structure social capital in promoting mental health in some literature. For example, after a detailed analysis of urban older adults, Lu revealed that cognitive social capital obviously increases self-rated health of respondents, but structural aspects do not (Lu and Zhang, 2019). Forsman and his colleagues discovered that both trust and social contact are beneficial to the improvements of depressive symptoms, however, former produces a greater effect (Forsman et al., 2012).

More importantly, the indirect effect of cognitive social capital might differ from that of structural social capital in the relationship. From the perspective of structural social capital, the Internet not only increases opportunities to interact with relatives (Hogeboom et al., 2010; Ruppel et al., 2016), but also promotes social participation such as attendance at clubs, volunteer work, and religious services (Kim et al., 2017), ultimately enhances older adults' mental health. Therefore, structural social capital might play a mediating role between Internet use and depressive symptoms.

As far as cognitive social capital is concerned, using Internet implies that individuals are easily exposed to misleading news, fraud, as well as various social scandals due to lack of effective information filtering (Sabatini and Sarracino, 2019). This prolonged exposure to online risks gradually shakes the confidence of participants in others and the authority (Guess et al., 2018), thus impairing their subjective well-beings. The adverse impact might be particularly common in older adults, because their cognitive abilities decline with the rising ages. Therefore, cognitive social capital may suppress the positive association between Internet use and mental health.

**Figure 1:**
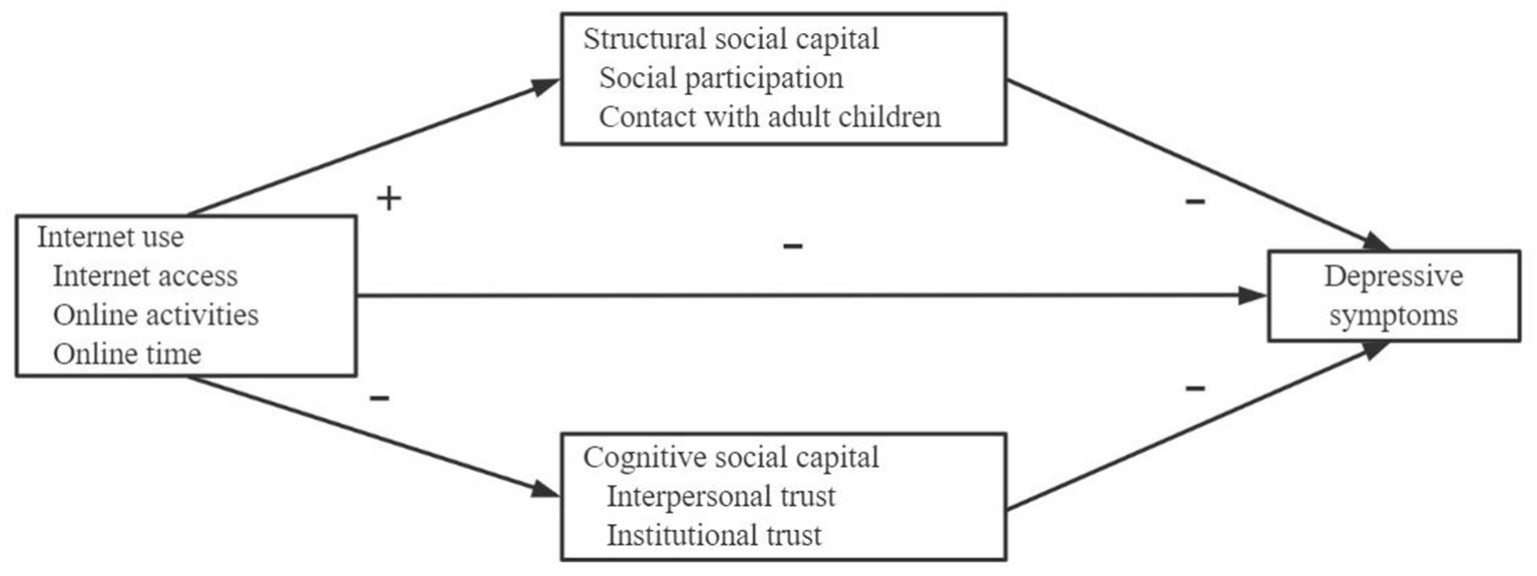
The theoretical model.

To address the above-mentioned gaps in previous literature, this study aims to further investigate the social mechanism between the Internet and depression among older adults. Taking into account the multiple dimensions of the Internet and social capital, this paper mainly discusses how various aspects of Internet use (i.e., Internet access, online activities, and online time) affect depressive symptoms through structural social capital (i.e., social participation and contact with adult children) and cognitive social capital (i.e., interpersonal trust and institutional trust). According to the literature review, a theoretical model is constructed (Figure 1) and the following hypotheses are put forward:

H1. Internet access, online activities, and online time are associated with reduced depressive symptoms in older adults.
H2. As social participation, contact with adult children, interpersonal trust, and institutional trust increase, the depressive symptoms in older adults decrease significantly.
H3. Internet use characteristics boost social participation and contact with adult children, thereby alleviating depressive symptoms; however, they impair interpersonal trust and institutional trust, which in turn exacerbate depression.

## Materials and Methods

### Research Data

This study used the Wave 5(2018) survey of the China Family Panel Studies (CFPS), which was initiated by Peking University. The baseline survey of CFPS was implemented in 2010 and the respondents were tracked every 2 years. Because of its comprehensive questionnaire, this survey vividly portrays the changes in Chinese lifestyle, ideology and social structure from 2010 to 2018 (Xie and Hu, 2014). Considering that the Internet gradually became popular among older adults in China in 2018, this study focused on the data in the Wave 5 of CFPS consisting of 32,669 respondents. After excluding observations with missing data and the data of respondents aged 59 and younger, the final sample in this study was restricted to 6,840 observations.

### Variables and Measurements

#### Depressive Symptoms

A cumulative indicator was designed to measure the degree of depressive symptoms. By a widely used scale (Briggs et al., 2018), the respondents reported the rate of depressed feelings such as insomnia, loneliness in the past week. Then we merged these responses into a new indicator, namely depressive symptoms (Cronbach's α = 0.83). The value of this variable ranged from 0 to 24. And the larger the value was, the more serious the depressive symptoms were.

#### Internet Use

Internet use was indicated by Internet access, online activities and online time. Internet access was based on two direct questions asked to respondents. The first one was “Do you get on the Internet by a computer?” and the other was “Do you use mobile devices, e.g., a mobile phone, to access the Internet?” Both of the responses were dichotomous (1 = Yes and 0 = No). As long as the participants had access to the Internet, this paper assigned a value of 1 to Internet access, with 0 in other cases.

#### Online Activities

Online activities were categorized into emotional activities and instrumental activities. The former was measured by the average frequency of Internet use for social and entertainment purposes, and the latter was assessed by the average frequency of Internet use for work and study purposes. Responses were from 1(never) to 7(almost everyday).

#### Online Time

Respondents were asked how long they spend online each week (0 = 0~7 h, 1 = 7~14 h, 2 = 14~21 h, 3 = more than 21 h).

#### Structural Social Capital

Social participation was measured with a binary variable. The older adults were asked whether they attended Labor union, religious groups or Association of workers (0 = None, 1 = at least one type); Contact with adult children was indicated by the frequency of communicating with adult children in the last 6 months. Responses were from 1 (never) to 7 (almost everyday).

#### Cognitive Social Capital

Interpersonal trust could be assessed by an average score of 2 responses, which asked respondents to rate the degree of trust in neighbors and in strangers. Institutional trust was assessed by an average score of trusting in local government and doctors. Responses were from 0 (distrustful) to 10 (very trustworthy).

#### Control Variables

Certain personal traits and socioeconomic variables were employed in the empirical analysis. Age was measured in years; Marital status was divided into two categories (0 = Unmarried and 1 = Married); Education attainment referred to the highest education level of the respondents. It was divided into four groups: illiterate, primary school, junior high school, and senior high school and above, ranging from 0 to 3. Gender was coded into a binary variable (1 = Male and 0 = Female). Hukou, namely Chinese household registration system, was measured by a dichotomous variable (1 = Urban residents and 0 = Rural residents). Living conditions referred to whether the respondents lived alone (0 = Living alone and 1 = Living with others). Relative income level was assessed by self-rated income level of respondents, where “1” represented the lowest income and “5” represented the highest income. Living arrangements referred to whether the older adults lived with their children (0 = No and 1 = Yes).

### Analytical Strategy

Given that the dependent variable was a continuous variable, we employed stepwise ordinary least square (OLS) regressions-using *reg* commend in Stata-to test the hypothesis 1 and hypothesis 2. In the first step, age, marital status, education attainment, gender, living conditions and relative income level were entered as control variables. Then, indicators of Internet use and social capital were added into the models in turn. The variance inflation factors (VIFs) values of the variables included in this study were all <1.57, which avoided the problem of multicollinearity.

In order to test the pathways between Internet use and depressive symptoms, generalized structural equation modeling (GSEM)-“gsem” commend in Stata-was used to confirm the hypothesis 3. In GSEM, responses can be continuous or binary, ordinal, count, or multinomial, which better adapted to our study data. Only AIC and BIC values of the models were presented due to no other goodness of fix indexes for GSEM.

## Results

**Figure 2:**
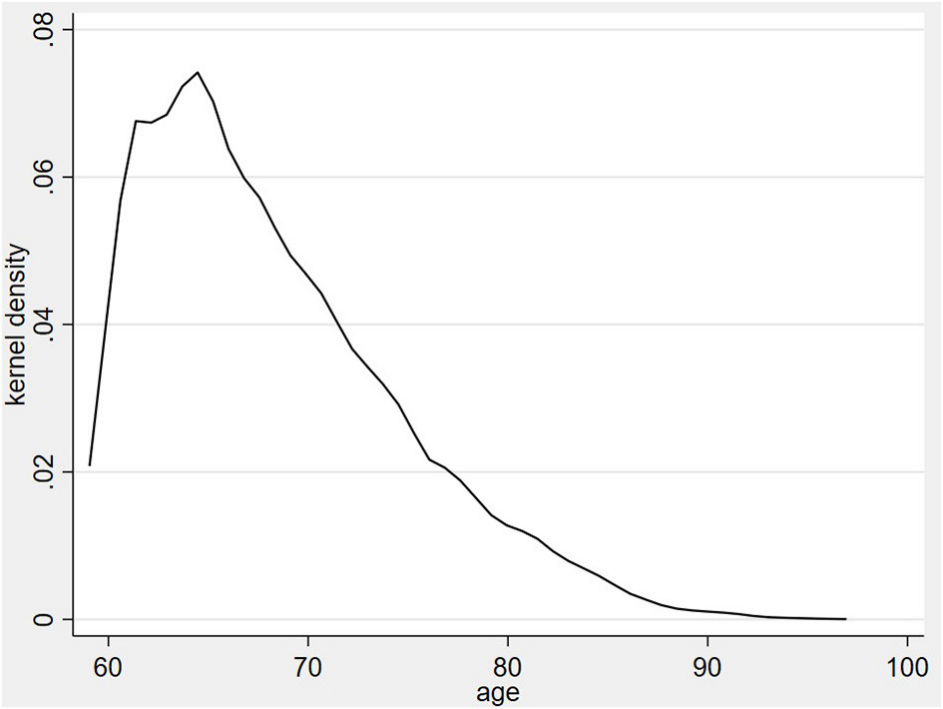
Age distribution of the respondents.

Table 1 reports the basic information of the respondents. Depressive symptoms averaged 5.72. Nearly 13.1% of the respondents had access to the Internet. Older adults commonly used the Internet for emotional purposes rather than instrumental purposes. Concerning structural social capital, 16% of the older adults attended at least one social organization, and the average frequency of interaction with adult children was 3.20. With regard to cognitive social capital, the average of interpersonal trust was 4.48, which was slightly lower than that of institutional trust (6.34). The older adults in general were undereducated. Specifically, almost 89% of them had junior high school or below. <40% of older adults lived with their children. Figure 2 shows the age distribution of the sample, which was basically in line with the age structure of Chinese older adults in 2018.

**Table 1:** Descriptive statistics (*N* = 6,840).

**Variable**	**Mean/%**	**Std. Dev**.	**Min**.	**Max**.
Depressive symptoms	5.72	4.46	0	24
Internet use				
Internet access	13.1%	–	–	
Emotional activities	1.49	1.47	1	7
Instrumental activities	1.13	0.64	1	7
Online time	0.16	0.58	0	3
Structural social capital				
Social participation	15.89%	–	–	–
Contact with adult children	3.20	2.10	0	6
Cognitive social capital				
Interpersonal trust	4.48	1.69	0	10
Institutional trust	6.34	2.26	0	10
Age	67.96	6.17	60	95
Gender	51.33%	–	–	–
Marriage	85.72%	–	–	–
Hukou	30.51%	–	–	–
Education				
Illiteracy	44.74%	–	–	–
Primary school	24.69%	–	–	–
Junior high school	18.93%	–	–	–
Senior high school and above	11.64%	–	–	–
Living conditions	93.39%	–	–	–
Relative income level	3.04	1.19	1	5
Living arrangements	35.38%	–	–	–

Table 2 shows the results from the OLS regressions for depressive symptoms. As indicated in Model (1), male, married and urban residents showed lower levels of depression, and so did people with higher education attainment. Living with others was negatively associated with depressive symptoms. Relative income level contributed to maintaining mental health, whereby a 1-unit increase in income level resulted in a ~0.4-unit drop in depressive symptoms.

**Table 2:** Depressive symptoms regressed on Internet use and social capital.

	**(1)**	**(2)**	**(3)**	**(4)**	**(5)**	**(6)**	**(7)**
Age	−0.01	−0.01	−0.01	−0.01	−0.01	−0.01	−0.01
	(0.01)	(0.01)	(0.01)	(0.01)	(0.01)	(0.01)	(0.01)
Gender	−1.05^***^	−1.05^***^	−1.08^***^	−1.09^***^	−1.09^***^	−1.08^***^	−1.09^***^
	(0.11)	(0.11)	(0.11)	(0.11)	(0.11)	(0.11)	(0.11)
Marriage	−1.12^***^	−1.11^***^	−1.09^***^	−1.07^***^	−1.08^***^	−1.07^***^	−1.08^***^
	(0.17)	(0.17)	(0.17)	(0.17)	(0.17)	(0.17)	(0.17)
Hukou	−1.20^***^	−1.14^***^	−1.11^***^	−1.18^***^	−1.16^***^	−1.22^***^	−1.19^***^
	(0.12)	(0.13)	(0.13)	(0.13)	(0.13)	(0.13)	(0.13)
Education							
Primary school	−0.85^***^	−0.84^***^	−0.77^***^	−0.77^***^	−0.77^***^	−0.79^***^	−0.78^***^
	(0.13)	(0.13)	(0.13)	(0.13)	(0.13)	(0.13)	(0.13)
Junior high school	−0.95^***^	−0.91^***^	−0.83^***^	−0.86^***^	−0.85^***^	−0.89^***^	−0.87^***^
	(0.15)	(0.15)	(0.15)	(0.15)	(0.15)	(0.15)	(0.15)
Senior high school	−1.45^***^	−1.34^***^	−1.26^***^	−1.24^***^	−1.22^***^	−1.31^***^	−1.26^***^
	(0.19)	(0.20)	(0.20)	(0.19)	(0.19)	(0.19)	(0.19)
Living conditions	−0.93^***^	−0.93^***^	−0.93^***^	−0.96^***^	−0.95^***^	−0.95^***^	−0.95^***^
	(0.25)	(0.25)	(0.25)	(0.24)	(0.24)	(0.24)	(0.24)
Relative income level	−0.44^***^	−0.44^***^	−0.44^***^	−0.36^***^	−0.36^***^	−0.35^***^	−0.35^***^
	(0.04)	(0.04)	(0.04)	(0.04)	(0.04)	(0.04)	(0.04)
Living arrangements	−0.22*	−0.23*	−0.24*	−0.23*	−0.23*	−0.23*	−0.23*
	(0.11)	(0.11)	(0.11)	(0.11)	(0.11)	(0.11)	(0.11)
Internet use							
Internet access		−0.47^**^	−0.36^*^	−0.41^*^			
		(0.17)	(0.17)	(0.17)			
Emotional activities					−0.11^**^		
					(0.04)		
Instrumental activities						−0.10	
						(0.08)	
Online time							−0.20^*^
							(0.10)
Structural social capital							
Social participation			0.20	0.20	0.20	0.20	0.20
			(0.14)	(0.14)	(0.14)	(0.14)	(0.14)
Contact with adult children			−0.15^***^	−0.14^***^	−0.14^***^	−0.15^***^	−0.14^***^
			(0.03)	(0.03)	(0.03)	(0.03)	(0.03)
Cognitive social capital							
Interpersonal trust				−0.15^***^	−0.15^***^	−0.15^***^	−0.15^***^
				(0.03)	(0.03)	(0.03)	(0.03)
Institutional trust				−0.16^***^	−0.16^***^	−0.16^***^	−0.16^***^
				(0.02)	(0.02)	(0.02)	(0.02)
Intercept	10.59^***^	10.75^***^	11.82^***^	12.80^***^	12.95^***^	12.77^***^	12.74^***^
	(0.70)	(0.71)	(0.72)	(0.73)	(0.73)	(0.73)	(0.73)
*N*	6,840	6,840	6,840	6,840	6,840	6,840	6,840
adj. *R*^2^	0.0959	0.0964	0.1011	0.1132	0.1136	0.1127	0.1130

*Standard errors in parentheses,*

* *p < 0.05,*

** *p < 0.01,*

*** *p < 0.001*.

The indicators of Internet use were sequentially entered into Model (2) to Model (7). Apparently the older adults who used Internet tended to have less depressive symptoms than those who did not access the website. Furthermore, online emotional activities and the intensity of Internet use were both negatively correlated with depressive symptoms. However, instrumental purposes, such as online study and work, were not associated with mental health. Our first hypothesis (H1) is partially supported.

The indicators of social capital were added into Model (3) to Model (7). Then we found that social participation did not work on mental health in older adults, which seems beyond our inference. Yet, contact with adult children significantly reduced depressive symptoms. Model (4) presents the positive effects of cognitive social capital on mental health, where both interpersonal trust and institutional trust predicted the significant decline in depressive symptoms. Our second hypothesis (H2) is partially confirmed.

Moreover, we also noticed that the influence of Internet access on depressive symptoms in Model (2) tended to diminish in Model (3), and its coefficient changed from −0.47 to −0.36. This implies the link between Internet use and depressive symptoms was partially mediated by the indicators of structural social capital. After addition of interpersonal trust and institutional trust in Model (4), the absolute value of the coefficient of Internet access increased by 14% compared with model (3), which suggested indictors of cognitive social capital were likely to act as the suppressing role. Therefore, GSEM was performed to further test the mediating effect and suppressing effect of social capital.

**Figure 3:**
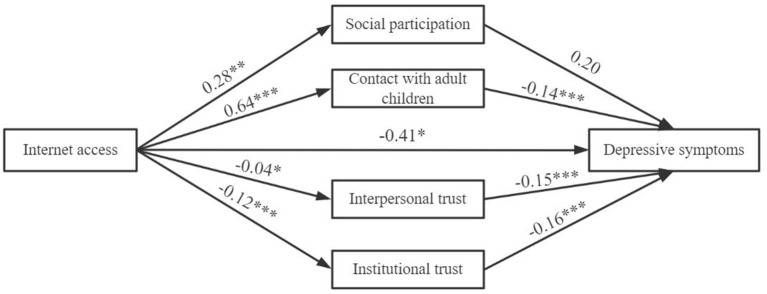
The structural model of the effect of Internet access on depressive symptoms. *p < 0.05, **p < 0.01, ***p < 0.001. The confounding variables had been strictly controlled. LL = −38,187.79, DF = 26, AIC = 65,503.6, BIC = 42,087.6.

**Figure 4:**
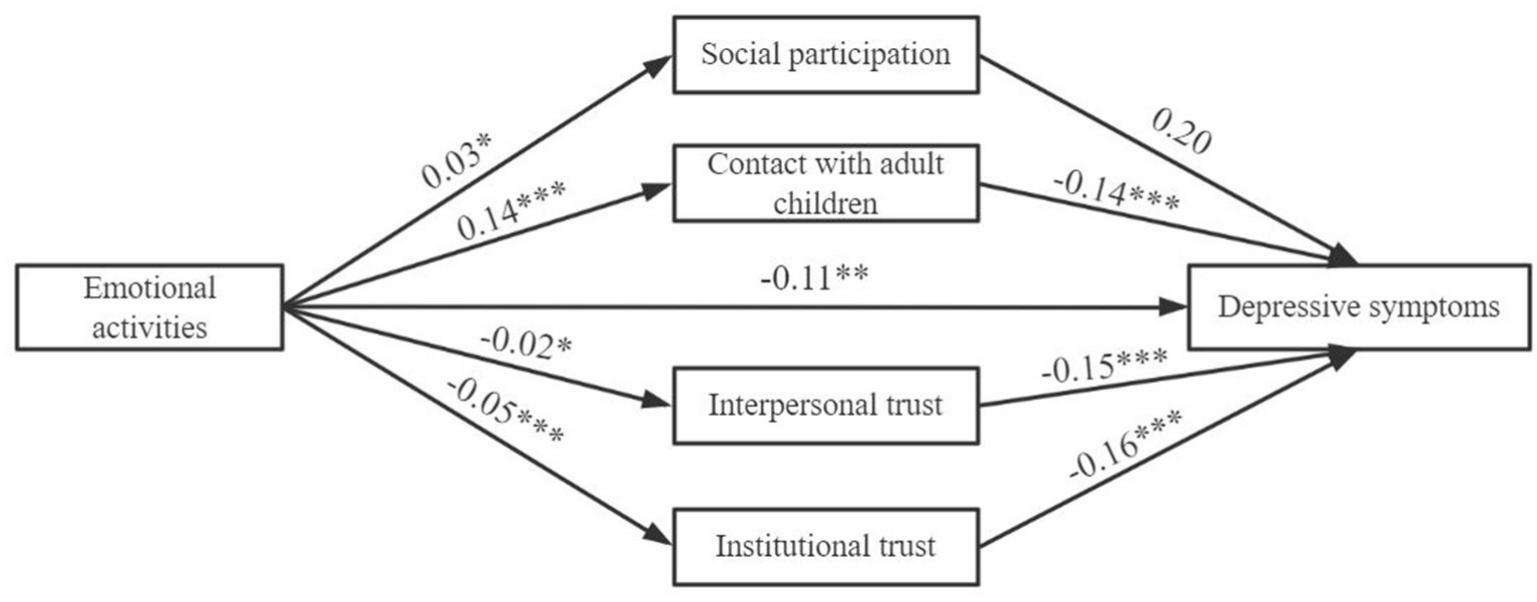
The structural model of the effect of emotional activities on depressive symptoms. *p < 0.05, **p < 0.01, ***p < 0.001. The confounding variables had been strictly controlled. LL = −43,957.56, DF = 26, AIC = 43,237.3 BIC = 54,059.7.

**Figure 5:**
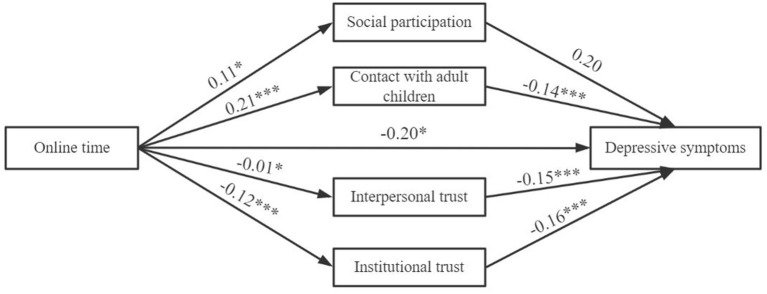
The structural model of the effect of online time on depressive symptoms. *p < 0.05, ***p < 0.001. The confounding variables had been strictly controlled. LL = −46,583.98, DF = 26, AIC = 47,458.7, BIC = 43,705.2.

Figures 3–5 display that each dimension of Internet use was not only directly related to depressive symptoms, but also exerted an indirect influence through social capital. However, the nature of the indirect effects of social participation and trust were different.

For structural social capital, Internet use predicted a significantly higher odds of social participation, but social participation was unrelated to depressive symptoms. Meanwhile, we discovered that Internet use facilitated contact with adult children, and this increase in frequency of the interaction was negatively related to depressive symptoms. Considering that the direction of direct effect of Internet use on depressive symptoms was consistent with that of its indirect effect on depressive symptoms by contact with adult children, contact with adult children could be recognized as a mediating factor in the relationship (MacKinnon et al., 2000).

For cognitive social capital, Internet use indicators impaired the respondents' interpersonal trust and institutional trust. For instance, Older adults who used the Internet decreased by 0.04-unit in interpersonal trust and 0.12-unit in institutional trust than those without Internet access. This aggregate decline of cognitive social capital resulted in more severe depressive symptoms. In view of the fact that Internet use directly improved mental health, but at the same time it indirectly aggravated depression by reducing trust, therefore, cognitive social capital could be regarded as a suppressor. Our third hypothesis (H3) is supported.

We employed a bootstrapping method to test whether the indirect effects were statistically significant (1,000 resamples). The significance of indirect effects depends on whether the 95% confidence intervals include zero. Table 3 reports that the indirect effects of Internet use predicting depressive symptoms by social participation were not significant, while the mediation pathways of contact with adult children were significantly negative. For components of cognitive social capital, Internet use had positive indirect effects on depressive symptoms by interpersonal and institutional trust. Based on bootstrapped indirect effects and direct effects, we discovered that indicators of social capital partially accounted for the impact of Internet use on depressive symptoms.

**Table 3:** Mediation and suppression effects.

**Pathway**	**Observe**	**Indirect effect**
	**coefficient**	**with 95% CI**
Internet access		
IA->SP->DEPS	0.057	[−0.007, 0.106]
IA->CAC->DEPS	−0.090	[−0.114, −0.067]
IA->IPT->DEPS	0.006	[0.061, 0.104]
IA->INT->DEPS	0.018	[0.006, 0.029]
Emotional activities		
EA->SP->DEPS	0.006	[−0.024, 0.039]
EA->CAC->DEPS	−0.020	[−0.031, −0.008]
EA->IPT->DEPS	0.003	[0.011, 0.023]
EA->INT->DEPS	0.007	[0.001, 0.006]
Online time		
OT->SP->DEPS	0.023	[−0.037, 0.085]
OT->CAC->DEPS	−0.029	[−0.046, −0.015]
OT->IPT->DEPS	0.002	[0.022, 0.064]
OT->INT->DEPS	0.019	[0.003, 0.016]

*IA, Internet access; EA, emotional activities; OT, online time; SP, social participation; CAC, contact with adult children; IPT, interpersonal trust; INT, institutional trust; DEPS, depressive symptoms*.

## Discussion

Using the 2018 CFPS survey, this study examined the underlying mechanisms between Internet use, social capital and depressive symptoms in Chinese settings. In line with hypothesis 1, after controlling for demographic variables, we found that Internet access, emotional activities and screen time could effectively reduce depressive symptoms, in support of the previous findings (Cotten et al., 2014). Nevertheless, we did not discover a significant association between online instrumental activities and mental health. Potential reasons may be attributed to the functional disparities of various online activities and the influence of family culture on the behaviors of older adults. From the use and gratifications perspective, online emotional activities can strengthen private relationships, providing emotional support and improving life satisfaction, and online instrumental activities are regarded as a functional participation that helps to enhance self-identity and obtain useful information. On these grounds, we can make a preliminary inference that the effect of Internet use on mental health among older Chinese adults mainly depends on affective regulation and emotional support, rather than information acquisition and instrumental value.

Partly consistent with hypothesis 2, our results showed interaction with adult children and trust might predict a significant decline in depressive symptoms. However, current study failed to observe a negative effect of social participation in depressive symptoms, which contradicts the findings that social participation helps to relieve depression (Wang W. et al., 2019). A possible explanation for this divergence is the defect of our measurement of social participation. Due to restrictions on data, social participation in current study only involved religious groups, labor union, and association of workers, all of which are not common activities for Chinese older adults. Thus, we did not have sufficient samples to reject the null hypothesis.

Hypothesis 3 has been verified. To our best knowledge, these findings furnish the first evidence that social capital has a dual role, carrying mediation and suppression effects in the link between Internet use and mental health.

In terms of structural social capital, the Internet could break the constraints of space and physical inactivity for older adults (Oishi, 2010), and establish more positive and time-efficient interactions than face-to-face communication (Mellor et al., 2008; Sum et al., 2008b). This remote communication is important for Chinese older adults to keep mental health. For one thing, Industrialization and urbanization have prompted increasing adult children to leave their hometowns, leading to old age empty-nest families. For another, private family relationships construct older adults' life meaning and self-esteem in Confucianism culture (Wang W. et al., 2019). Thus, using Internet to enhance the interactions with their adult children is of great practical value and theoretical significance for older adults to mitigate depression.

In terms of cognitive social capital, the Internet might bring blurred information for older adults, which exerts a bad influence on their behaviors and attitudes including trust. The potential reason is that negative and misleading contents in websites would lead to a decline in recognition of shared values and undermine the deference to authority (Im et al., 2014), then threaten older adults' trust. This decrease of interpersonal trust and institutional trust represents a pessimistic view (e.g., insecurity and anxiety) on their living environment, exacerbating mental health problems in older adults. Therefore, the positive association between Internet use and mental health is suppressed by cognitive social capital.

Besides, it is notable that the indirect effect of contact with adult children was greater than that of trust, which might be interpreted by the notion of the socioemotional selectivity theory (STT). The SST suggests that actual time left in life, to some extent, determines relative importance of different life events (Carstensen, 2006). For younger people, they prioritize long-term goals such as obtaining new information and achieving personal value. For older adults, they prioritize short-term events such as interacting with families and gaining emotional support, rather than expanding horizons. In consequence, the beneficial influence of increased contact with adult children caused by internet use was much stronger compared to the adverse effect of decreased trust on mental health in older adults.

This study had several limitations. First, although GSEM was conducted to examine how the Internet affects depressive symptoms by social capital, endogeneity may still exist due to cross-sectional data. Second, there was a deficiency in the validity of the measurement of social participation. Under the constraints of data, social activities such as square dancing and mahjong that Chinese older adults are passionate about were not reflected in the research. Third, since the role of internet use may be different for older adults with diagnosed depressive disorders, we identified the older adults with depression by cut-off point widely used in previous studies (Briggs et al., 2018). And then we found that the relationship between Internet use and depressive symptoms was robust regardless of depression among older adults. Nonetheless, the measurement of depressive symptoms was based on self-reported scores rather than medical diagnosis, which may pose a threat to the accuracy of mental health assessment. Fourth, compared with Internet access or online time, a more nuanced insight of internet use, such as WeChat and Tiktok use, is necessary for future studies. Therefore, more detailed data should be employed to deepen our research.

## Conclusion

Taken together, this study provides fresh evidence for the limited studies on the health implications of the Internet in older adults. Our results displayed the Internet could promote structural social capital, which in turn reduced depressive symptoms. Meanwhile, Internet use also destroyed older adults' cognitive social capital, then consequently exacerbated depressive symptoms. This dual role of social capital indicates that the mechanism through which Internet affects depressive symptoms is complicated and diverse. Therefore, in order to promote older adults' mental health in a low-cost way, we call for not only improvement in network infrastructure construction, but also related Internet training courses for older adults.

## Data Availability Statement

Publicly available datasets were analyzed in this study. This data can be found at: http://www.isss.pku.edu.cn/cfps/sjzx/gksj/index.htm.

## Ethics Statement

The studies involving human participants were reviewed and approved by Peking University. The patients/participants provided their written informed consent to participate in this study.

## Author Contributions

MY: conceptualization and methodology. ZL: data analysis and writing. Both authors contributed to the article and approved the submitted version.

## Conflict of Interest

The authors declare that the research was conducted in the absence of any commercial or financial relationships that could be construed as a potential conflict of interest.

## Publisher's Note

All claims expressed in this article are solely those of the authors and do not necessarily represent those of their affiliated organizations, or those of the publisher, the editors and the reviewers. Any product that may be evaluated in this article, or claim that may be made by its manufacturer, is not guaranteed or endorsed by the publisher.
